# Construction of a Super-Competent *Bacillus subtilis* 168 Using the P_*mtlA*_-*comKS* Inducible Cassette

**DOI:** 10.3389/fmicb.2015.01431

**Published:** 2015-12-21

**Authors:** Regine Rahmer, Kambiz Morabbi Heravi, Josef Altenbuchner

**Affiliations:** Institut für Industrielle Genetik, Universität StuttgartStuttgart, Germany

**Keywords:** DNA uptake, transformation rate, competence, mannitol induction, *comK*

## Abstract

Competence is a physiological state that enables *Bacillus subtilis* 168 to take up and internalize extracellular DNA. In practice, only a small subpopulation of *B. subtilis* 168 cells becomes competent when they enter stationary phase. In this study, we developed a new transformation method to improve the transformation efficiency of *B. subtilis* 168, specially in rich media. At first, different competence genes, namely *comK, comS*, and *dprA*, were alone or together integrated into the chromosome of *B. subtilis* 168 under control of mannitol-inducible P_*mtlA*_ promoter. Overexpression of both *comK* and *comS* increased the transformation efficiency of *B. subtilis* REG19 with plasmid DNA by 6.7-fold compared to the wild type strain 168. This transformation efficiency reached its maximal level after 1.5 h of induction by mannitol. Besides, transformability of the REG19 cells was saturated in the presence of 100 ng dimeric plasmid or 3000 ng chromosomal DNA. Studying the influence of global regulators on the development of competence pointed out that important competence development factors, such as Spo0A, ComQXPA, and DegU, could be removed in REG19. On the other hand, efficient REG19 transformation remained highly dependent on the original copies of *comK* and *comS* regardless of the presence of P_*mtlA*_-*comKS*. Finally, novel plasmid-free strategies were used for transformation of REG19 based on Gibson assembly.

## Introduction

Since the first report on transformation of *Bacillus subtilis* by Spizizen (1958), the competency of *B. subtilis* has intensively been studied revealing highly interconnected regulatory networks (Hamoen et al., 2003). These interconnected regulatory networks are responsible for fine-tuning of the single-cell and social behavior of *B. subtilis* in a culture medium, specially during their entrance to stationary phase. When *B. subtilis* cells enter stationary phase due to nutrient deprivation and high cell density, they start to differentiate into various subpopulations. Some of them become motile (Nishihara and Freese, 1975), while the others form biofilm (Vlamakis et al., 2008), secrete degradative enzymes and antibiotics (González-Pastor et al., 2003), or finally sporulate (Rudner and Losick, 2001; Piggot and Hilbert, 2004). Another small subpopulation differentiates into competent cells able to take up extracellular DNA (Dubnau, 1991a; Dubnau and Provvedi, 2000).

**Figure 1:**
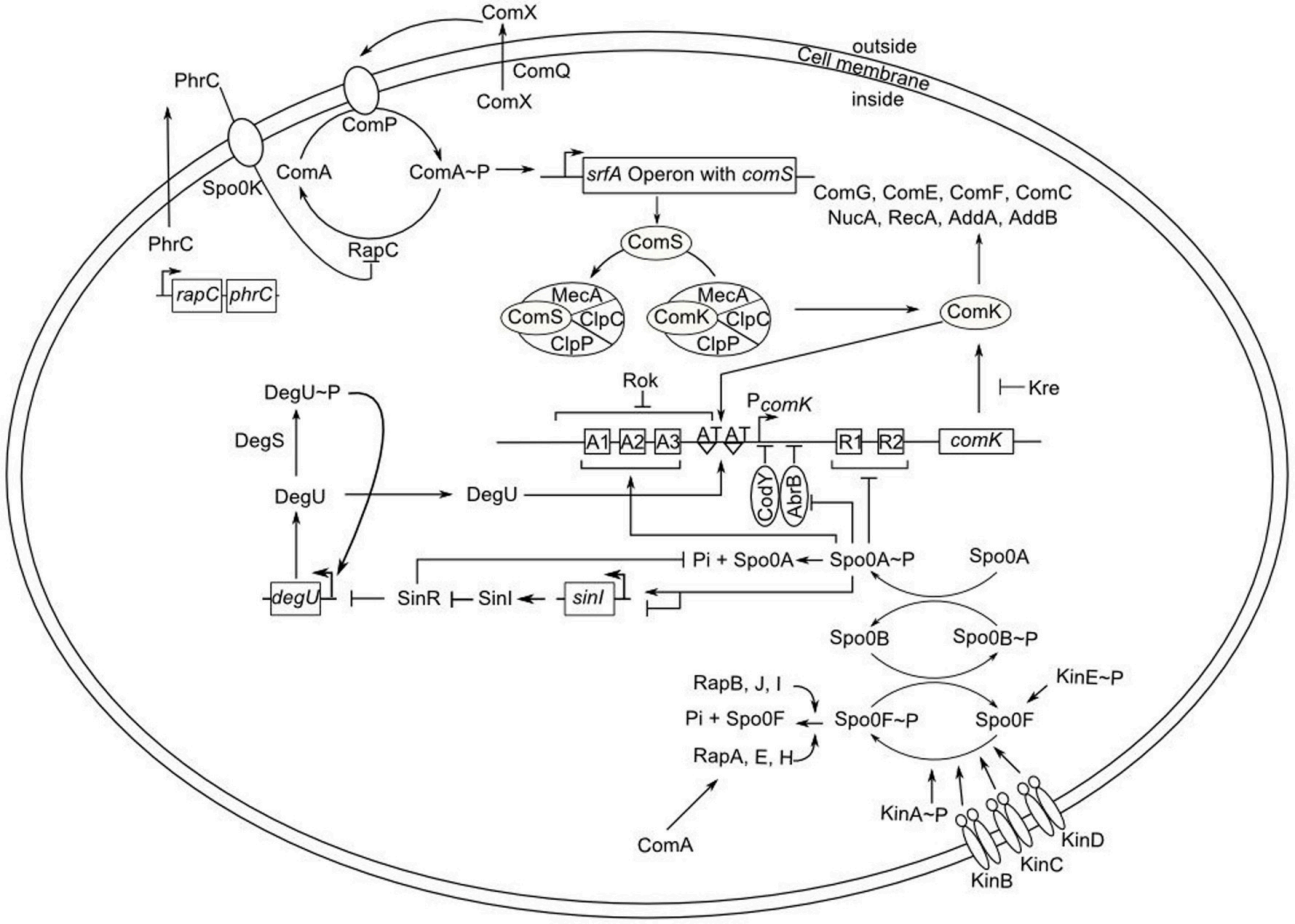
**Overview of competence regulation in ***B. subtilis*****. Lines ending in perpendiculars and arrows denote negative and positive effects, respectively. The quorum sensing mechanism containing the modified peptide ComX results in phosphorylation of the ComA response regulator by ComP. ComA~P activates the transcription of the *srf* operon with the embedded *comS* gene. *ComS* binds to the protease complex of MecA, ClpC, and ClpP and prevents the degradation of the transcription factor ComK. The stability of the *comK* mRNA is also influenced by Kre. The transcription of *comK* is regulated by various factors. ComK activates its own expression by binding to the AT-boxes in its promoter region. This binding is facilitated by DegU. At low concentration, Spo0A~P binds to three special sequences (A1–A3; high affinity binding sites) in the promotor of *comK*. At high concentration, Spo0A~P is capable of binding to further sequences (R1 and R2; low affinity binding sites) and repressing the expression of *comK*. Spo0A~P also release the *comK* expression by repressing AbrB. Other repressing factors of the *comK* expression are CodY and Rok. Rap proteins, such as RapA, prevent ComA~P interaction with its target DNA and dephosphorylate Spo0F~P. The expression of *sinI* is activated at the low level of Spo0A~P and repressed by a high level of Spo0A~P concentration. SinI antagonizes SinR, which represses the expression of *degU*. Finally, high concentration of ComK activates the expression of genes for DNA-uptake and -integration as well as many other genes.

Generally, the main actor in *B. subtilis* differentiation processes is Spo0A, the transcriptional master regulator of sporulation. Spo0A is phosphorylated via a complex phosphorelay system consisting of five histidine kinases (KinA, B, C, D, and E), a phosphate acceptor protein (Spo0F) and a phosphotransferase (Spo0B) (Burbulys et al., 1991). Depending on the level of phosphorylated Spo0A, the cells individually enter different states. Specifically, *B. subtilis* becomes competent when the competence transcription factor, ComK, reaches a certain threshold level (Maamar and Dubnau, 2005; Smits et al., 2005). ComK is the competence master regulator which activates about 100 genes for DNA-recombination, -repair, -binding, -uptake (Berka et al., 2002; Hamoen et al., 2002), cell division (Hamoen, 2011), as well as its own promoter in a positive feedback loop (van Sinderen and Venema, 1994). Therefore, the ComK pool in the cell is tightly regulated at the transcriptional level by the antagonizing global regulators AbrB (the transition state regulator) and Spo0A, CodY, and its specific regulator Rok (Hoa et al., 2002), as well as the DegS–DegU two component system (Hamoen et al., 2000; Figure 1).

During exponential phase, the expression of *comK* is repressed by Rok, AbrB, and by CodY, a pleiotropic repressor responding to the GTP level in the cell (Serror and Sonenshein, 1996). Recently, it has been found that the stability of *comK* mRNA is also influenced by Kre (YkyB) (Gamba et al., 2015; Figure 1). Moreover, the ComK pool in the cell is regulated at the posttranslational level. ComK is targeted by the adaptor protein MecA and degraded in a complex with ClpC and ClpP (Kong and Dubnau, 1994; Turgay et al., 1997). At the end of the exponential phase, changing the levels of DegU and phosphorylated Spo0A drastically alter the situation enabling the expression of *comK*. Briefly, DegU is phosphorylated by DegS in response to unknown intracellular signals. While phosphorylated DegU regulates the synthesis of degradative enzymes, biofilm formation, and capsule synthesis, DegU binds to the *comK* promoter region, thereby activates the transcription of *comK* (Dahl et al., 1992; Figure 1). Moreover, phosphorylated Spo0A represses the expression of *abrB*, releasing *comK* expression from repression by AbrB (Hahn et al., 1995). Therefore, Δ*spo0A* strains are poorly transformable (Sadaie and Kada, 1983; Albano et al., 1987). In a concerted manner with the relief of *comK* from repression, a small protein, ComS, is able to protect ComK from the ClpC-ClpP-MecA complex during the stationary phase (Turgay et al., 1998; Prepiak et al., 2011; Figure 1). The *comS* gene is located within the *srf* open reading frame encoding surfactin and its expression is regulated by quorum sensing signaling pathway provoked by the ComQXPA cluster. ComX, an extracellular pheromone modified by ComQ, is sensed by a membrane-bound sensor histidine kinase (ComP), eventually resulting in the activation of its cognate response regulator ComA (Magnuson et al., 1994; Comella and Grossman, 2005). Phosphorylated ComA induces expression of *comS* and activates natural competence (Hahn and Dubnau, 1991; Figure 1).

When ComK molecules reach a sufficient amount and the cells become competent, they are able to take up DNA. On the surface of a competent cell, the double-stranded DNA binds to a receptor via a pseudopilus similar to type IV pili, cleaved by a membrane-localized nuclease and internalized as a linear single stranded DNA (ssDNA). Inside the cell, ssDNA is protected by single strand DNA binding proteins, such as DprA, SsbA, SsbB, and RecN (Inamine and Dubnau, 1995). During transformation, DprA protects the DNA inside the cell and is more important during the transformation with plasmid than with chromosomal DNA (Tadesse and Graumann, 2007). Moreover, DprA facilitates the displacement of SsbA and SsbB and mediates the attachment of RecA to ssDNA, which has already been covered by Ssb proteins (Yadav et al., 2013). Finally, the ssDNA is integrated into the genome via homologous recombination by RecA (Kidane et al., 2012).

Despite all the theoretical progress, *B. subtilis* is routinely transformed using a minimal medium according to the 50 years old method described by Anagnostopoulos and Spizizen (1961), albeit with slight modifications. This method is a time-consuming and inefficient method for transformation, specially for natural *B. subtilis* isolates (Duitman et al., 2007). To improve transformation efficiency, several attempts were carried out with regulated expression of *comK* as well as *comS* (Hahn et al., 1996; Liu et al., 1996; Duitman et al., 2007; Nijland et al., 2010; Zhang and Zhang, 2011). In this work, we expressed *comK, comS*, and *dprA* alone or in combined artificial operons under control of the *B. subtilis* mannitol promoter (P_*mtlA*_). In this way, we studied the influence of other important factors like ComQXPA, DegU, and Spo0A on transformation efficiency of plasmids and chromosomal DNA in these strains. Thereby, we constructed a strain with high transformation efficiency in rich medium. To our knowledge, this is the first attempt for overexpression of two competence factors in combination.

## Materials and methods

### Strains, media, and growth condition

All bacterial strains used in this study are listed in Table 1. *Escherichia coli* JM109 was used for plasmid construction and propagation. *E. coli* strain NM538 was used for multimerization of the monomer plasmids. All *E. coli* transformants were selected on LB agar (Bertani, 1951) supplemented with ampicillin (100 μg ml^−1^) or spectinomycin (100 μg ml^−1^) depending on the plasmid marker. When plasmids were used for transformation of *B. subtilis*, transformants were selected on LB agar containing kanamycin (10 μg ml^−1^), spectinomycin (100 μg ml^−1^), erythromycin (5 μg ml^−1^), or chloramphenicol (5 μg ml^−1^). The tryptophan or histidine auxotroph mutants were cultivated on Spizizen's minimal medium (Anagnostopoulos and Spizizen, 1961) supplemented with 50 μg ml^−1^ tryptophan or histidine, respectively. *B. subtilis* strains transformed with chromosomal DNA of strain KM0 (*trp*^+^) were selected on Spizizen's minimal medium containing glucose (0.5% w/v).

**Table 1:** **Strains used in this study**.

**Strain**	**Genotype**	**Source, Reference, or Construction**
***E. coli***		
JM109	*recA1, endA1, gyrA96, thi-1, hsdR17*($r_{K}^{-}$, $m_{k}^{+}$), *mcrA, supE44, gyrA96, relA1, λ^−^, Δ(lac-proAB)*, F′ (*traD36, proAB^+^, lacI^*q*^, lacZΔ*M15)	Yanisch-Perron et al., 1985
NM538	*supF, hsdR, trpR, lacY*	Frischauf et al., 1983
***B. subtilis***		
168	*trpC2*	DSM23778^*^
IIG-Bs168-1	*trpC2, ΔmanPA*::*ermC*	pJOE6577.1 → 168
KM0	*trpC*	pKAM041 → 168
REG1	*trpC2, ΔmanPA*::*ermC, hisI*′-*spcR*-*yvcA*	pHM30 → IIG-Bs168-1
REG3	*trpC2, ΔmanPA*::*ermC*, P*_*mtlA*_-comS*	pREG3 → REG1
REG4	*trpC2, ΔmanPA*::*ermC*, P*_*mtlA*_-dprA*	pREG4 → REG1
REG5	*trpC2, ΔmanPA*::*ermC*, P*_*mtlA*_-dprA-comS*	pREG5 → REG1
REG6	*trpC2, ΔmanPA*::*ermC*, P*_*mtlA*_-comK*	pREG6 → REG1
REG7	*trpC2, ΔmanPA*::*ermC*, P*_*mtlA*_-comK-dprA*	Gibson 1 → REG1
REG19	*trpC2, ΔmanPA*::*ermC*, P*_*mtlA*_-comK-comS*	pJOE7361.1 → REG1
REG32	*trpC2, ΔmanPA*::*ermC*, P*_*mtlA*_-comK-comS, ΔcomS*	pREG12 → REG19
REG33	*trpC2, ΔmanPA*::*ermC*, P*_*mtlA*_-comK-comS, ΔcomK*	pREG13 → REG19
REG35	*trpC2, ΔmanPA*::*ermC*, P*_*mtlA*_-comK-comS, ΔcomK, ΔcomS*	pREG13 → REG32
REG36	*trpC2, ΔmanPA*::*ermC*, P*_*mtlA*_-comK-comS, Δspo0A*	pJOE7122 → REG19
REG37	*trpC2, ΔmanPA*::*ermC*, P*_*mtlA*_-comK-comS, ΔcomQXPA*	pREG10 → REG19
REG120	*trpC2, ΔmanPA*::*ermC*, P*_*mtlA*_-comK-comS, ΔdegU*	pREG58 → REG19

* *Deutsche Sammlung von Mikroorganismen und Zellkulturen*.

### DNA manipulation

Oligonucleotides used in this study were synthesized by Eurofins MWG Operon (Ebersberg, Germany; Table S1). All plasmids used in this study are listed in Table 2. Standard molecular techniques were carried out as described before (Sambrook et al., 1989). Chemicals, enzymes, and kits used for this study are described in Supplementary Materials.

**Table 2:** **Plasmids used in this study**.

**Plasmid**	**Genotype**	**Source or Reference**
pHM30	*ori*_pUC18_, *bla*, ′*hisF*-*hisI*′-*spcR*-′*yvcA-yvcB*′	Motejadded and Altenbuchner, 2007
pHM31	*ori*_pUC18_, *bla*, ′*hisF-hisI*-′*yvcA-yvcB*′	Motejadded and Altenbuchner, 2007
pIC20R	*ori*_pUC18_*, bla, lacZα*	Marsh et al., 1984
pJOE2962	*ori*_*pACYC*184_, *cat, rhaP_*BAD*_*-*mrpA*	Warth et al., 2011
pJOE4370.7	*ori^+^*_pUB110_, *ori*_pUC18_, *rep*_pUB110_, *kanR, bla, ble*	Altenbuchner, unpublished
pJOE4786.1	*ori*_pUC18_, *bla, ter*-′*lacI-lacZα*-*ter*	Altenbuchner et al., 1992
pJOE6577.1	*ori*_pUC18_, *bla, spcR*, ′*manR*-*manP*-*ermC-yjdF*	Wenzel and Altenbuchner, 2015
pJOE6743.1	*ori*_pUC18_, *spcR, bla*, P*_*manP*_*-*manP*	Wenzel and Altenbuchner, 2015
pJOE6905.1	*ori*_pUC18_, *bla, ter*-P_*mtlA*_-*comK*-*ter*	This study
pJOE7122.1	*ori*_pUC18_, *spcR, bla*, P*_*manP*_*-*manP, yqiG*-*recN*′	This study
pJOE7331.2	*ori*_pUC18_, *bla*, P_*mtlA*_-*comK*-*comS*	This study
pJOE7361.1	*ori*_pUC18_, *bla, yvcB*′-P_*mtlA*_-*comK*-*comS*-*hisI-hisF*′	This study
pKAM041	*ori*_pUC18_, *bla, ter*-′*trpD*-*trpC*-*trpF*′-*ter*	This study
pKAM180	*ori*_pUC18_, *bla*, ′*hisF-hisI*-*ter*-*luc*-P_*mtlA*_-*ter*-′*yvcA-yvcB*′	This study
pREG3	*ori*_pUC18_, *bla, yvcB*′-P_*mtlA*_-*comS*-*hisI-hisF*′	This study
pREG4	*ori*_pUC18_, *bla, yvcB*′-P_*mtlA*_-*dprA*-*hisI-hisF*′	This study
pREG5	*ori*_pUC18_, *bla, yvcB*′-P_*mtlA*_-*dprA*-*comS*-*hisI-his*F′	This study
pREG6	*ori*_pUC18_, *bla, yvcB*′-P_*mtlA*_-*comK*-*hisI-hisF′*	This study
pREG10	*ori*_pUC18_, *spcR, bla*, P*_*manP*_*-*manP*,*′mrpG-degQ*	This study
pREG12	*ori*_pUC18_, *spcR, bla*, P*_*manP*_*-*manP, ′hxlR-ycxA′*	This study
pREG13	*ori*_pUC18_, *spcR, bla*, P*_*manP*_*-*manP, ′yhzC-yhxD′*	This study
pREG58	*ori*_pUC18_, *spcR, bla*, P*_*manP*_*-*manP*, ′*degS-yviA*′	This study
pWAL275	*ori^+^*_pUB110_, *ori*_pUC18_, *rep*_pUB110_, *kanR, bla, mrpS*	Warth and Altenbuchner, unpublished

### Construction of the integration plasmid carrying functional *trpC*

To facilitate the growth of *B. subtilis* 168 in minimal media, the *trpC2* gene was replaced with functional *trpC* using the pKAM041 integration plasmid. The deleted codon was added to the *trpC2* sequence using primary PCRs with s8475-s8634 and s8633-s8476 oligonucleotide pairs. The primary PCR products were then fused in a PCR using oligonucleotides s8475 and s8476. The final PCR product was then inserted into pJOE4786.1 cut with *Sma*I to create pKAM041.

### Construction of integration plasmids for inducible gene expression

To integrate the P_*mtlA*_-*comK* cassette into the *B. subtilis* chromosome, the P_*mtlA*_ DNA fragment was amplified in a PCR using oligonucleotides s6851 and s6853. In the second PCR, the *comK* gene together with its ribosomal binding site was amplified with oligonucleotides s6854 and s6852. The two primary PCR products overlapping each other were finally fused in a PCR with primers s6851-s6852. The P_*mtlA*_-*comK* DNA fragment was then inserted into the pJOE4786.1 plasmid via *Sma*I sites creating pJOE6905.1. Next, the P_*mtlA*_-*comK* cassette was cut out from pJOE6905.1 by *Bam*HI and inserted into pHM31 via the same restriction site to create pREG6. For generation of the P_*mtlA*_-*comK*-*comS* (or P_*mtlA*_-*comKS*) cassette, the P_*mtlA*_-*comK* DNA fragment was amplified from pJOE6905.1 in a PCR using oligonucleotides s6851 and s7707. Primers s7708 and s7709 were used to amplify the *comS* gene from *B. subtilis* 168 in the second PCR. Both P_*mtlA*_-*comK* and *comS* DNA fragments were fused in a PCR with oligonucleotides s6851 and s7709. The PCR product (P_*mtlA*_-*comKS*) was primarily inserted into pJOE4786.1 via *Sma*I sites (pJOE7331.2). The P_*mtlA*_-*comKS* cassette was then cut out from pJOE7331.2 by *Bam*HI and inserted into pHM31 through *Bam*HI sites creating pJOE7361.1.

Further integration plasmids for inducible gene expression were constructed based on pKAM180 (for construction see Supplementary Materials), another pHM31-derivative containing P_*mtlA*_-*luc* (luciferase) cassette. Both *comS* and *dprA* were amplified with their ribosomal binding site using oligonucleotide pair s8003-s8004 (*comS*) and s8005-s8006 (*dprA*), respectively, for creating the P_*mtlA*_-*comS* and P_*mtlA*_-*dprA* cassettes. Each PCR fragment was then inserted via *Afl*II and *Nhe*I into pKAM180 to create pREG3 (P_*mtlA*_-*comS*) and pREG4 (P_*mtlA*_-*dprA*). For generation of the P_*mtlA*_-*dpA*-*comS*, the amplified *comS* DNA fragment was cut by *Brs*GI and *Afl*II, whereas the PCR fragment of *dprA* by *Bsr*GI and *Nhe*I. Afterwards, both fragments were inserted into pKAM180 digested with *Afl*II and *Nhe*I to create pREG5. For generation of the P_*mtlA*_-*comK*-*dprA*, the amplified *dprA* DNA fragment with the ribosomal binding site was amplified using oligonucleotide pair s10576-s10577 with overlapping sequences to pREG6. pREG6 was cut by *Nhe*I and the PCR fragment of *dprA* integrated by Gibson Assembly as described below (Gibson 1).

### Construction of gene deletion plasmids

Deletion plasmids were mainly constructed based on pJOE6743.1 carrying spectinomycin resistance gene (selection marker) and P_*manP*_-*manP* (anti-selection marker). Each deletion cassette was created by ligation of the two flanking fragments (cut *Eco*RI and *Sph*I) of the target deletion region with pJOE6743.1 (cut *Sph*I) in a three fragment ligation. For the *comQXPA* deletion, the oligonucleotide pair s8125–s8126 and s8127–s8128 were used and the PCR fragments were inserted via *Sph*I and *Eco*RI (pREG10). For the *comS* deletion, oligonucleotides s8172–s8249 and s8250–s8175 were used and inserted via *Bam*HI and *Eco*RI (pREG12). Oligonucleotides s8251–s8132 and s8252–s8253 were used for the *comK* deletion and inserted via *Sph*I and *Eco*RI (pREG13). Deletion of *degU* was carried out using oligonucleotides s9314–s9315 and s9316–s9317 followed by the insertion of the PCR products via *Sph*I and *Eco*RI into pJOE6743.1 (pREG58). For the *spo0A*-*spoIVB* deletion, oligonucleotides s7328–s7329 and s7330–s7331 were used. The amplified fragments were fused in a PCR and inserted blunt ended between the *Sma*I sites of pJOE6743.1 (pJOE7122.1).

### Construction and multimerization of the pWAL275 plasmid

The pJOE4370.7 plasmid is a shuttle vector consisting of a 3.7 kb *Sca*I-*Pvu*II fragment of pUB110 inserted into the *Sma*I site of plasmid pIC20R. To create pWAL275, the *mrpS* site for site-specific recombination of plasmid SCP2 was inserted between *Aat*II and *Hind*III sites of pJOE4370.7 using two complementary oligonucleotides *mrpS*-A and *mrpS*-B. Transformation of *E. coli* JM109 containing the *mrpA*-expressing pJOE2962 with the pWAL275 monomers led to multimerization of pWAL275. Plasmid DNA was then isolated from JM109 pJOE2962 pWAL275 (chloramphenicol and ampicillin resistance) again and used to transform JM109 without pJOE2962 (ampicillin resistance). Finally, the transformants were screened for the pWAL275 dimers using agarose gel electrophoresis (Warth et al., 2011). In all experiments, the dimers of pWAL275 were used for transformation of *B. subtilis* strains.

### Bacterial transformation

Transformation of *E. coli* with plasmid DNA was done as described previously (Chung et al., 1989). The “Paris method” was used to transform *B. subtilis* strains in Spizizen's minimal medium as described before (Harwood and Cutting, 1990). To transform the constructed strains carrying competence genes (*comK, comS, dprA*) expressed by the *B. subtilis* mannitol promoter (P_*mtlA*_), a new transformation protocol was developed. A 5 ml overnight culture, inoculated from a single colony, with an OD_600_ of about 1.7 was used to inoculate shake flasks containing 10 ml LB with an OD_600_ of 0.1 and incubated at 37°C with 200 rpm. After 90 min incubation, 0.5% (w/v) mannitol was added and the bacterial culture was further incubated for 90 min. The cells were then washed in LB medium and diluted to an OD_600_ of 0.5. Unless otherwise stated, 100 ng of the pWAL275 dimers or 3 μg of chromosomal DNA of the KM0 strain were added to each 1 ml aliquots of the cell suspension. After adding the desired DNA, the cell suspension was incubated on a roller drum for 1 h at 37°C. Depending on the DNA, transformants were selected as described above.

### Construction of *B. subtilis* strains by markerless gene deletion/integration systems

In this study, two strains, KM0 (*trp*^+^) and IIG-Bs168-1 (*trpC2* Δ*manPA*), were constructed by direct transformation of *B. subtilis* 168 (*trpC2*). Primarily, strain 168 was transformed with pKAM041 in Spizizen's minimal medium containing tryptophan (50 μg ml^−1^) in order to replace the *trpC*2 mutation with the functional *trpC* gene. The 168 transformants with pKAM041 were selected on Spizizen's minimal medium without tryptophan. In this way, the tryptophan prototroph strain KM0 was constructed. On the other hand, transformation of strain 168 with pJOE6577.1 created strain IIG-Bs168-1, the parental host for the markerless gene deletion system. In IIG-Bs168-1, the *manPA* operon encoding mannose-specific PTS transporter and mannose 6-phosphate dehydrogenase were replaced by an erythromycin resistance gene. The *manPA* deletion was the essential modification for the markerless gene deletion method in *B. subtilis* using pJOE6743.1 derivatives (Wenzel and Altenbuchner, 2015). Markerless integration of the desired promoter-gene cassettes, such as P_*mtlA*_-*comK*, was carried out using the pHM30/pHM31 system based on histidine auxotrophy (Motejadded and Altenbuchner, 2007). Briefly, first the linearized plasmid pHM30 was integrated into the IIG-Bs168-1 (*his*^+^) genome via homologous recombination replacing the *hisI* gene with a spectinomycin resistance gene (strain REG1). In the second step, the *his*-auxotophic strain REG1 was transformed by a linearized suicide vector containing the *hisI* gene together with the P_*mtlA*_-gene cassette and selected for histidine-prototrophic and spectinomycin sensitive colonies.

### Gene deletion using cassettes constructed by Gibson assembly

Three PCR fragments were fused using the Gibson Assembly® Cloning Kit (New England BioLabs®, Frankfurt, Germany) as recommended by the manufacturer to generate a linear DNA cassette for disruption of the *amyE* gene in *B. subtilis* (encodes α-amylase). The upstream homolog fragment of *amyE* was amplified in a PCR using oligonucleotides s10446 and s10447, while the downstream homolog fragment was amplified with oligonucleotides s10450 and s10451. Between the amplified upstream and downstream flanking fragments, the spectinomycin resistance gene (*spcR*) was amplified with oligonucleotides s10448 and s10449. These three DNA fragments were assembled to create the deletion cassette. In a second strategy, the flanking fragments of the deletion cassette were amplified using oligonucleotides s10453–s10447 and s10450–s10452. These newly amplified flanking fragments contained 20 base pairs overlap at their outer ends enabling them to form multimers or circulated DNA fragments after Gibson assembly. After fragments assembly, 10 μl of each mixture (single linear vs. multimer linear and circular cassettes) were used for REG19 transformation.

## Results

### Induction of competence by insertion of *comK* under control of p_*mtlA*_

**Figure 2:**
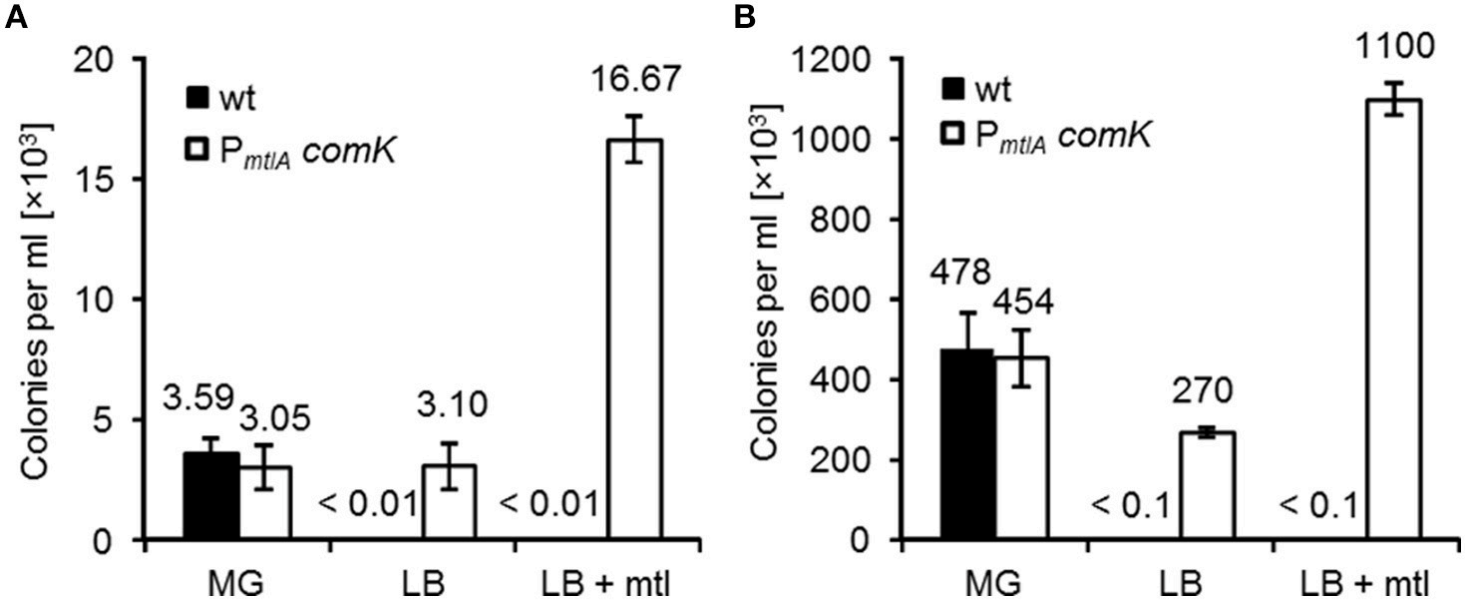
**Transformation efficiency of ***B. subtilis*** 168 (wt) and REG6 (P_***mtlA***_-***comK***)**. Cells were transformed according to Paris method using Spizizen's minimal medium containing glucose (MG) and the new protocol using LB medium with(out) mannitol. 100 ng of the pWAL275 dimers **(A)** and 3 μg of KM0 chromosomal DNA **(B)** were used for transformation. Error bars represent standard deviation from the mean value of triple experiments.

ComK is the master regulator of competence in *B. subtilis*. In practice, competence in *B. subtilis* is induced in minimal medium by quorum sensing signals and nutritional stress. After extensive growth in an amino acid rich medium, the cells are shifted to an amino acid deprived medium for a short time to induce competence in a two-step procedure (Paris method). To render competence independent of the growth medium and to enhance the transformation efficiency, the *comK* gene was inserted under control of the *B. subtilis* mannitol promoter (P_*mtlA*_) and integrated into the chromosome of *B. subtilis* 168 (strain REG6). Likewise, a new protocol with Luria–Bertani (LB) medium was developed to transform REG6. Briefly, an overnight culture was diluted in LB and incubated for 90 min at 37°C on a rotary shaker. Afterwards, mannitol was added at a final concentration of 0.5% and the culture was further incubated for 90 min. Cells were harvested, washed and resuspended in 1 ml aliquots. DNA was then added and cells were plated on selective agar plates after 1 h incubation at 37°C. To compare the transformation efficiency between *B. subtilis* 168 (wt) and REG6, the Paris method and the new protocol were used. The wt and REG6 strains were transformed using either pWAL275 dimers (a pUB110-pUC18 derivative) or chromosomal DNA of a *trpC*^+^ derivative of *B. subtilis* 168 (strain KM0). In the case of pWAL275, the transformed cells were selected on LB with kanamycin, whereas in the case of chromosomal DNA, the transformed cells were selected on Spizizen's minimal agar plates without tryptophan. The results are shown in Figures 2A,B. There was no significant difference between transformation efficiency of the wt and REG6 using plasmid DNA (Figure 2A) or chromosomal DNA (Figure 2B) when the Paris method with Spizizens' minimal glucose (or MG medium) was used. In LB medium without mannitol, very few kanamycin resistant and *trpC*^+^ colonies were obtained with the wild type strain. In strain REG6, the number of transformants was significantly increased in LB. When REG6 cells in LB were induced with mannitol, the number of transformants was increased by about 2.3-fold compared to the Paris method using chromosomal DNA and 4.6-fold using plasmid DNA. Transformation under DNA saturation condition with chromosomal DNA was about 100-fold more efficient than with plasmid DNA.

### Enhancing transformation efficiency by induction of *comK, dprA*, and *comS*

**Figure 3:**
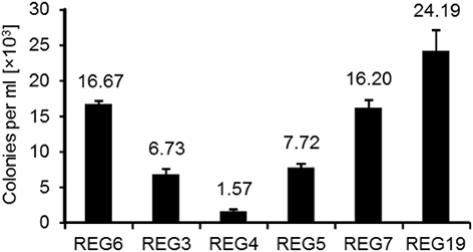
**Overexpression of different competence genes and their effect on transformation efficiency**. Strains *B. subtilis* REG6 (P_*mtlA*_-*comK*), REG3 (P_*mtlA*_-*comS*), REG4 (P_*mtlA*_-*dprA*), REG5 (P_*mtlA*_-*dprA*-*comS*), REG7 (P_*mtlA*_-*comK-dprA*), and REG19 (P_*mtlA*_-*comK-comS*) were transformed with 100 ng pWAL275 dimers in LB with mannitol as described in Section Materials and Methods. Error bars represent standard deviation from the mean value between triple experiments.

Induction of *comK* by mannitol resulted in a higher transformation efficiency; however, competence development depends on more factors than ComK. For example, ComS controls degradation of ComK, and DprA conveys incoming ssDNA to RecA. To investigate the effect of ComS and DprA on transformation efficiency, *comS* and *dprA* alone, together or combined with *comK* were inserted downstream of P_*mtlA*_ and integrated into the *B. subtilis* 168 chromosome. The new strains were called REG3, REG4, REG5, REG7, and REG19. To compare these strains with REG6, all strains were grown in LB, induced with mannitol and transformed with pWAL275 dimer DNA (Figure 3). Induction of *comS* (REG3) and *dprA* (REG4) alone increased the number of transformants in comparison with the wt, albeit less efficient than REG6. The concomitant expression of *dprA* and *comS* (REG5) as well as *dprA* and *comK* (REG7) showed more transformants than with REG4 even though the number of transformed cells remained less than with REG6. Interestingly, REG19 with *comK* and *comS* combined in an artificial operon was superior to all other strains. The plasmid transformation frequency of REG19 was about 6.7-fold higher in comparison with wt transformed in MG medium. Hereafter, transformation efficiency of REG19 was studied in more detail.

### Optimization of the transformation protocol

**Figure 4:**
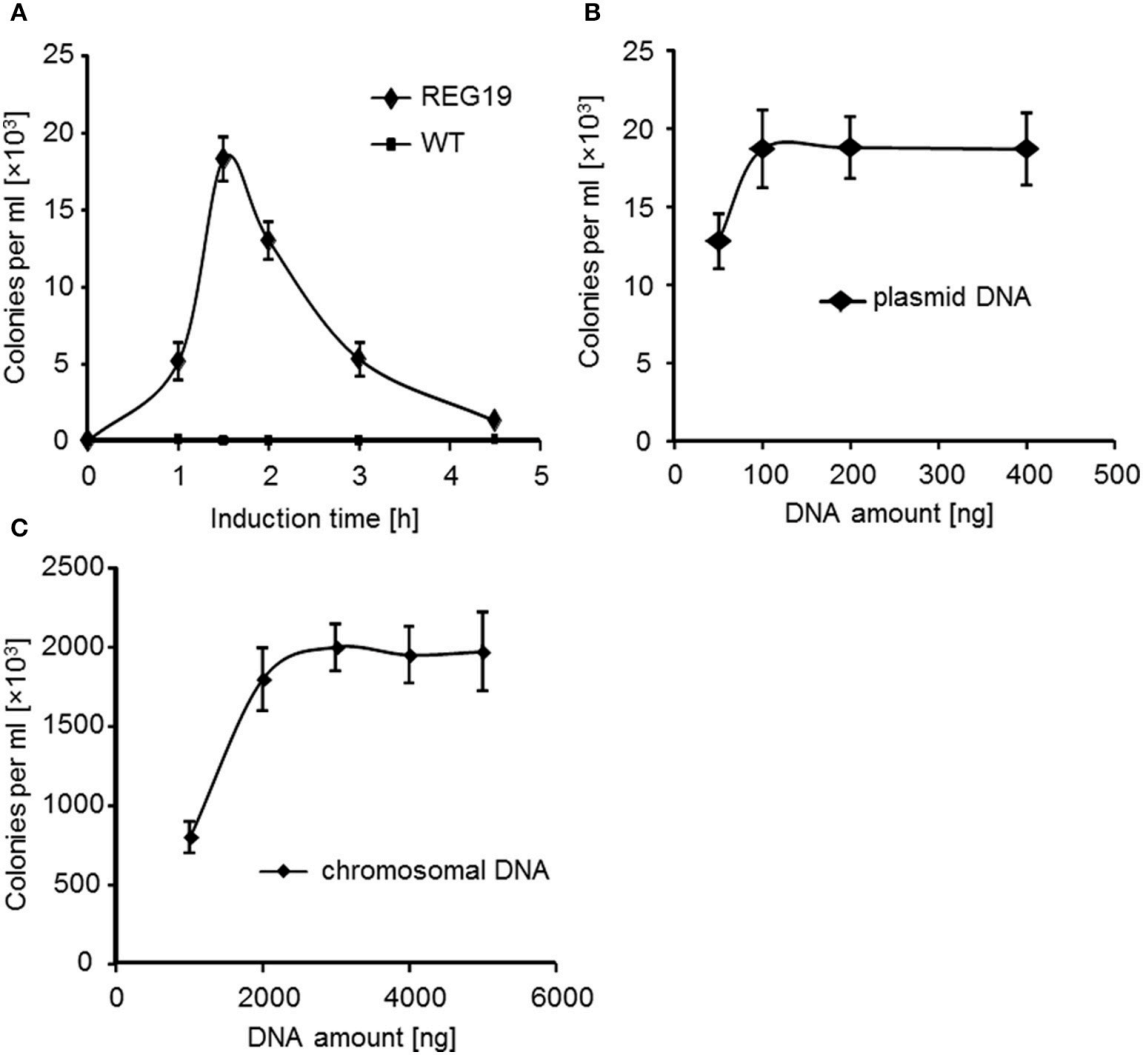
**Transformation efficiency of REG19 in LB medium with mannitol under different conditions. (A)** Transformation of strains 168 (wt), as a control, and REG19 was carried out with 100 ng pWAL275 dimers after different time of induction with mannitol. **(B)** Transformation efficiency of REG19 was also studied after 1.5 h of induction with 50–400 ng of dimer plasmid DNA or **(C)** 1000–5000 ng of chromosomal DNA. Error bars represent standard deviation from the mean value between triplicate experiments.

As noted above, the simultaneous induction of *comK* and *comS* resulted in the strongest transformation efficiency of *B. subtilis*. To see, whether the growth condition could be further optimized for competence development, the time of induction with mannitol was varied. As before, an overnight culture of the REG19 strain in LB was diluted in fresh LB, incubated in shake flasks for 1.5 h at 37°C and 0.5% (w/v) mannitol was added. Cultures were grown for 1, 1.5, 3, or 4.5 h to allow the induction of competence. The induced cultures were washed and diluted to an OD_600_ of 0.5 in 1 ml LB. pWAL275 DNA was added and the cultures were further incubated at 37°C for 60 min. Finally, dilutions were plated on LB agar containing kanamycin and the number of transformants counted at the next day. As shown in Figure 4A the highest number of transformants was achieved after 1.5 h of induction. Induction shorter or longer than 1.5 h decreased the transformation efficiency.

Variable DNA samples and amount, e.g., 50–400 ng DNA of the pWAL275, were added to REG19 in order to determine the transformation efficiency (Figure 4B). The correlation between transformation efficiency and DNA amount showed that there is DNA saturation point at 100 ng of dimer plasmid DNA. At DNA saturation condition, 19 × 10^3^ kanamycin resistant colonies per 1 ml competent cells were obtained with 100 ng pWAL275 dimer. 100 ng of the pWAL275 dimer equals 8 × 10^9^ DNA molecules. Since the OD_600_ of 0.5 corresponds to 2.5 × 10^8^ cells, the proportion of plasmid molecules to cells needed for achieving the maximal transformation rate is 32-fold for the dimer.

Further transformation experiments were done with REG19 and chromosomal DNA of *B. subtilis* KM0, by selection of the *trpC*^+^ transformants. In this case, saturation of transformation was obtained at about 3 μg chromosomal DNA (Figure 4C). Compared to plasmid DNA, the number of transformants was about 100- to 200-fold higher (2 × 10^6^ cells).

### The importance of original *comS* and *comK* in REG19

**Figure 5:**
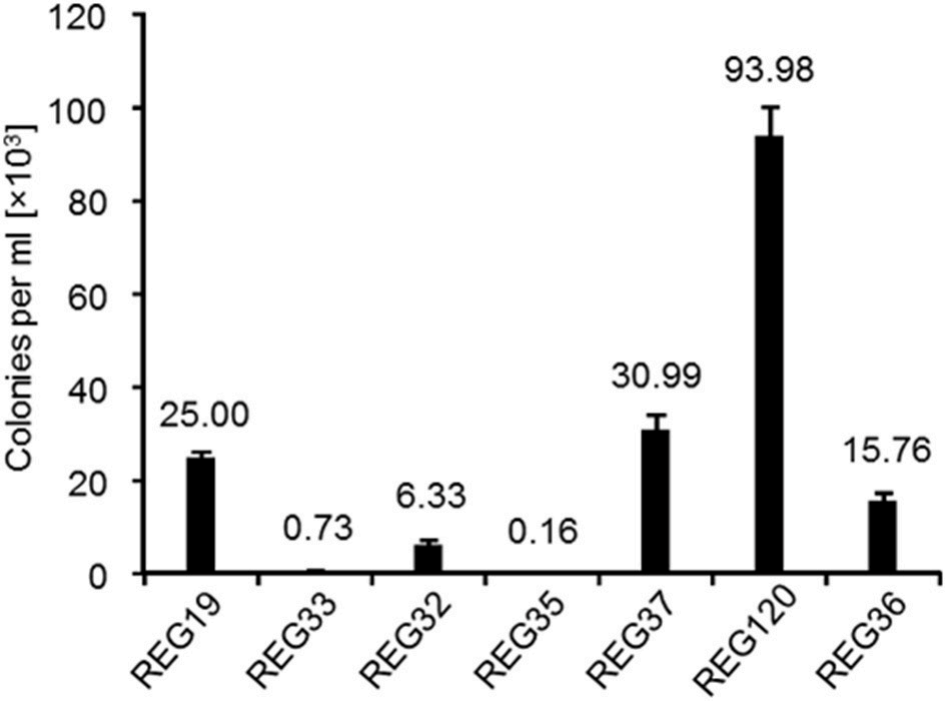
**Deletion of the competence development genes in REG19 (control) constructing REG32 (Δ***comS***), REG33 (Δ***comK***), REG35 (Δ***comK*** Δ***comS***), REG36 (Δ***spo0A***), REG37 (Δ***comQXPA***), REG120 (Δ***degU***), and their effect on transformation efficiency**. All strains were transformed in LB with 100 ng of pWAL275 dimers after induction with mannitol. Error bars represent standard deviation from the mean value between triplicate experiments.

So far, we have shown that the induction of combined *comK* and *comS* (REG19) results in high transformation efficiency of *B. subtilis*. To understand whether the synthesis of ComK and ComS driven by P_*mtlA*_ can bypass the requirements for regular competence-inducing factors, original *comK* and *comS* were deleted in REG19. For these experiments, strains REG33 (REG19 Δ*comK*), REG32 (REG19 Δ*comS*) and REG35 (REG19 Δ*comK* Δ*comS*) were constructed. Deletion of the native *comK* gene resulted in a 50-fold decrease in transformation efficiency. Obviously, *comK* expressed by P_*comK*_ remained necessary for effective transformation. Deletion of both *comK* and *comS* (REG35) drastically decreased the transformation rate (Figure 5). Although, *comS* seemed to be less important, deletion of the original *comS* in REG19 reduced the number of REG32 transformants by four-fold compared to REG19 (Figure 5). Since, natural expression of *comS* depends on the ComP-ComA signal transduction system, the *comQXPA* genes were deleted in REG19 (REG37). Surprisingly, deletion of the *comQXPA* slightly increased the transformation efficiency of REG37 as compared to REG19 (Figure 5). Apparently, the expression of original *comK* and *comS* are necessary for a high transformation efficiency of REG19.

### The effect of global regulators DegU and Spo0A on transformation efficiency of REG19

At the onset of competence development, when ComK concentration is still insufficient for activation of its own gene, the non-phosphorylated response regulator DegU facilitates binding of ComK to P_*comK*_ (Hamoen et al., 2000). In contrast, phosphorylated DegU leads away from competence and activates swarming motility, biofilm formation and protease secretion (Kobayashi, 2007; Verhamme et al., 2007). To investigate the necessity of DegU for competence development in REG19, *degU* was deleted in REG19 (REG120). In REG120, the transformation efficiency was nearly quadrupled in comparison to REG19 (Figure 5). Another important factor for competence is Spo0A (Sadaie and Kada, 1983; Albano et al., 1987). Phosphorylated Spo0A limits the intracellular level of AbrB, which represses the transcription of *comK*. Mirouze et al. (2012) showed that a low level of Spo0A~P induces *comK* expression, whereas its high level reduces the *comK* expression. In REG36 (REG19 Δ*spo0A*), the transformation efficiency was slightly reduced, from 25 × 10^3^ colonies per ml in REG19 to 15 × 10^3^ colonies per ml in REG36 (Figure 5). Obviously, the ectopic expression of *comK* and *comS* can compensate the need of Spo0A~P in the *comK* expression by its own promoter, while DegU is not further necessary for the onset of *comK* expression.

### Deletion of chromosomal genes in REG19 using Gibson assembly

**Figure 6:**
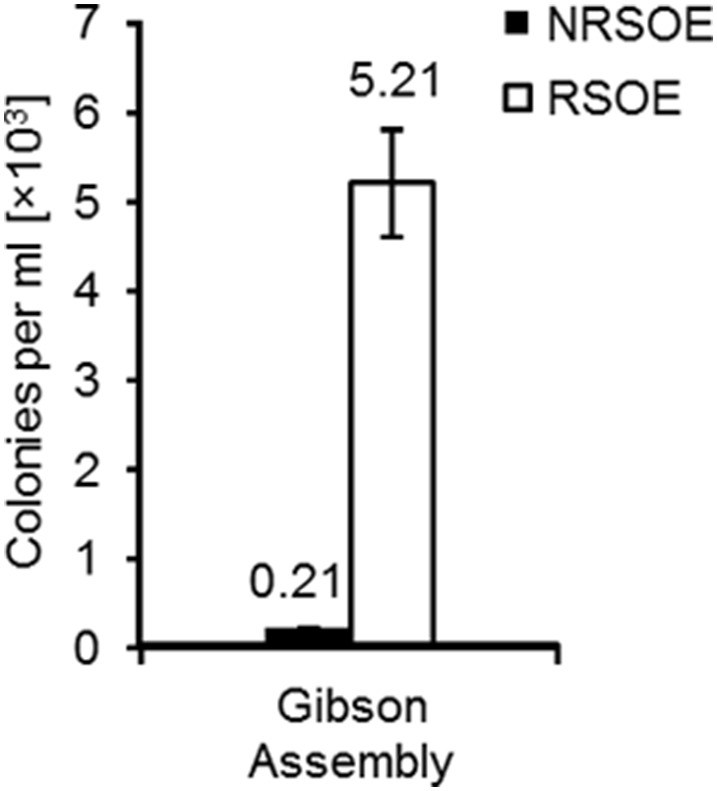
**Deletion of ***amyE*** in REG19 using linear or circular DNA fragments created by Gibson assembly**. The *amyE* deletion cassette consisted of two 700 bp flanking fragments homolog to the up-and downstream of *amyE* and a spectinomycin resistance gene in between. While NRSOE represents the single deletion cassettes generated by Gibson assembly, RSOE deletion cassettes were the mixture of long concatemer or circular deletion cassettes due to the overlapping regions at the both ends of each deletion cassette. After transformation of REG19, deletion of the *amyE* was verified in the spectinomycin resistant colonies by cultivation on starch agar. Error bars represent standard deviation from the mean value between triplicate experiments.

A usual way for deletion of target chromosomal DNA is its replacement with an antibiotic resistance gene via the homologous recombination. Since assembling a deletion cassette on a plasmid is time-consuming, such deletions could be accomplished after direct fusion of the amplified fragments followed by transformation of the host strain with high transformation efficiency. To demonstrate the feasibility of this method in REG19, the *amyE* gene was deleted using two types of ′*amyE*-*spcR*-*amyE*′ cassettes, which were assembled by Gibson assembly. In the first type, the end of the deletion cassettes could not overlap each other (No Repeated Sequences at the Outer Ends; NRSOE), whereas in the second type, each cassette contained 20 bp overlapping ends enabling the formation of concatemers or circular DNA fragments (Repeated Sequences at the Outer Ends; RSOE). After transformation of REG19 with NRSOE, about 200 spectinomycin resistant colonies per ml were obtained (Figure 6). All spectinomycin resistant colonies were amylase-negative on starch agar plates. Using RSOE fragments, there was a 25-fold increase in the number of spectinomycin resistant colonies compared to NRSOE fragments. This increased efficiency could be due to protection of the cassettes ends from nuclease digestion. Likewise, these overlapping ends allowed the assembly of cassettes to form larger linear fragments. This improves the uptake of single-stranded DNA which is long enough for efficient recombination. Despite the higher transformation efficiency, only 80% of the spectinomycin resistant transformants of NRSOE were amylase negative. This could be caused by circular cassettes integrated into the chromosome via single cross-over. Overall, high transformation rate of REG19 facilitates deletion of the target gene using assembled deletion cassettes without construction of plasmids.

## Discussion

In this study, we constructed a super competent *B. subtilis* strain (strain REG19) whose transformability is widely independent of the natural competence development. The natural competence development in *B. subtilis* is generally categorized into three types of regulations, namely (i) nutritional, (ii) cell-type, and (iii) growth stage specific (Dubnau, 1991b). In rich media, nutritional regulation is exerted by CodY repressing the promoters of *comK* and *comS* in the presence of branched-chain amino acids and high GTP pool (Serror and Sonenshein, 1996; Figure 1). Here, by insertion of *comK* under control of P_*mtlA*_, the *B. subtilis* cells became transformable in LB (strain REG6; Figure 2). Expressing *comK* with its natural promoter (van Sinderen and Venema, 1994), P_*spac*_ (Nijland et al., 2010), or P_*xylA*_ (Zhang and Zhang, 2011) similarly enabled the transformation of *B. subtilis* in rich media. The cell-type specific regulation or *comK* heterogenous expression in different cells is caused by autoregulatory loop of *comK* (Maamar and Dubnau, 2005). Surprisingly, deletion of original *comK* (REG33) or *comS* (REG32) in the REG19 strain had a severe impact on transformation (Figure 5). This suggests that the amount of expressed ComK and ComS by P_*mtlA*_ is insufficient to pass the ComK threshold stimulating competence development. Only when P_*mtlA*_ was exchanged with stronger P_*licB*_ (REG89), the cells were no longer dependent on original *comK* (compare REG89 with REG159 in Supplementary Materials; Figure S1).

The growth stage-regulation mainly depends on the quorum sensing in a minimal medium with high cell density. To eliminate this regulation, we also overexpressed *comS* in addition to *comK* which dramatically increased the transformation efficiency (compare strains REG6 and REG19 in Figure 3). This suggests that the expression of *comS* remained a barrier for the full transformability of the cells in most of developed systems so far. Although, the ectopic expression of *comK/comS* increased the transformability and rendered competence free of its natural development process, the natural competence regulators remained effective on the REG19 transformability. Notably, the phenotypes of the mutants were in some cases different from the same deletions in the wild type strain. For instance, a slight reduction of the transformation efficiency was observed in REG36 (REG19 Δ*spo0A*) compared to REG19 (Figure 5). In a low concentration of Spo0A(~P), the *comK* expression is increased by Spo0A(~P) binding to its high affinity binding sites at the *comK* promoter, where it antagonizes the binding of Rok. On the other hand, high Spo0A(~P) concentration represses *comK* expression when Spo0A(~P) binds to its low-affinity binding sites overlapping the P_*comK*_ core elements (Mirouze et al., 2012; Figure 1). Apart from its direct influence on P_*comK*_, Spo0A(~P) downregulates the AbrB expression, thereby it renders P_*comK*_ free of AbrB repression (Smits et al., 2007). Besides, high level of the AbrB concentration represses the expression of *sigH* which is important for the expression of *phr* genes. In the latter case, the DNA binding ability of ComA(~P) is influenced by the Spo0A deletion (Auchtung et al., 2006). Surprisingly, deletion of *comQXPA* in REG19 (strain REG37; Figure 5) had a slightly positive effect on transformability. In the wild type strain, this deletion could result in reduction or loss of transformability (Hahn and Dubnau, 1991); however, ComA(~P) regulates many genes other than *comS* (Ogura et al., 2001). Thus, the observed positive effect of the *comQXPA* deletion could be an indirect effect. The same argument might be true for the positive effect of *degU* deletion. The high level of transformability in REG120 could be explained by the possibility that the *degU* deletion eliminates routes of differentiation contrary to competence, such as flagellar based motility (Amati et al., 2004), biofilm formation as well as protease secretion (Msadek et al., 1990; Dahl et al., 1992; Kovács and Kuipers, 2013). This deletion supports the conclusion that ComK and ComS synthesized via P_*mtlA*_ compensate dephosphorylated DegU.

In conclusion, a super competent *B. subtilis* strain was constructed in this study by induction of *comK* and *comS* under control of P_*mtlA*_. The high transformability of this strain allows fast strain engineering using PCR fragments assembled by Gibson assembly kit. This transformation system is being successfully used for genome reduction of *B. subtilis* (Altenbuchner, unpublished data).

## Author contributions

RR designed and performed the experiments and also analyzed the data. KM constructed some of the plasmids and strains used in this study. Also, KM was involved in data analysis and manuscript preparation. JA directed and coordinated the project. The final paper was written by RR and KM which was later revised and corrected by JA. All authors approved the final version of the manuscript.

### Conflict of interest statement

The authors declare that the research was conducted in the absence of any commercial or financial relationships that could be construed as a potential conflict of interest.
