# Supplementary material for: Construction of a Super-Competent Bacillus subtilis 168 Using the PmtlA-comKS Inducible Cassette
Source: Front Microbiol. 2015 Dec 21;6:1431. doi: 10.3389/fmicb.2015.01431 (PMC4685060; doi:10.3389/fmicb.2015.01431)
Supplement: Supplementary file 1 [file DataSheet1.PDF]

Supplementary Material

Construction of a super-competent *Bacillus subtilis* 168 using the P_{mtlA}-*comKS* inducible cassette

Authors: Regine Rahmer, Kambiz Morabbi Heravi* and Josef Altenbuchner

Affiliation:

Institut für Industrielle Genetik, Universität Stuttgart, Allmandring 31, 70569 Stuttgart, Germany

***Corresponding author:** Kambiz Morabbi Heravi

E-Mail: kambiz.morabbi@iig.uni-stuttgart.de

Tel.: +49 711 685 66982

Fax: +49 711 685 66973

Materials and Methods

Table S1. Oligonucleotides used in this study.

Name	Sequence (5' → 3')	Purpose
s6851	GCTAGCTTTTTATTTTTAAAAAAT	Integration of P_{mtlA}
s6852	GCTATGTGACATCTCAGGT	Integration of *comK*
s6853	CCTCCATCCTTTTTCTGCACTTAAGAAATATGTTCAGAGAA	Integration of P_{mtlA}
s6854	TTCTCTGAACATATTTCTTAAGTGCAGAAAAAGGATGGAGG	Integration of *comK*
s7328	GGGATAAAGAATTGGATTATC	Deletion of *spo0A-spoIVB*
s7329	CATGAGCTTATTAAGTGGTC	Deletion of *spo0A-spoIVB*
s7330	GACCACTTAATAAGCTCATGACTGATGAACGAATGGATGT	Deletion of *spo0A-spoIVB*
s7331	GAGATTGACCAAATTGAAAAC	Deletion of *spo0A-spoIVB*
s7355	AAAAAAACCGGTTTTTTATTTTTAAAAAATTGTCACAGTCA	P_{mtlA} (pKAM114)
s7356	AAAAAAGGATCCTTTGTTATTGTCTGCCATATGGAATTCCTCC TTTAATTCTTAAGAAATATGTTCAGAGAATGATGC	P_{mtlA} (pKAM114)
s7705	AACATATGGAAGACGCCAAAAACAT	Luc
s7706	AAAAGCTTACGTATTACAATTTGGACTTTCCGC	Luc
s7707	CCTATTTTTCTAATACCGTTC	Integration of P_{mtlA}-*comK*
s7708	GAACGGTATTAGAAAAATAGGTGTATTTGGAAGAGAACGAAA	Integration of *comS*
s7709	GGAGGATTTCGTGCCGG	Integration of *comS*
s8003	AAAAACTTAAGCGAAAGCAAGGAGGAGCAGACG TTTGAACCGATCAGGCAAGCA	Integration of *comS*
s8004	AAAAAGCTAGCTGTACACTACTTCTCCCTCCAGCAG	Integration of *comS*
s8005	AAAAACTTAAGTGTACACAAAAGGAGGTCAA TCTATTGGATCAGGCCGCTGTCT	Integration of *dprA*
s8006	AAAAAGCTAGCTCAGGAAGTTCCTCGAAAATAT	Integration of *dprA*
s8125	AAAAAGCATGCATGCTGCTTCTAAAGGCTCAA	Deletion of *comQXPA*
s8126	AAAAAGAATTCATGTTGGGGGGTGTAGAGAT	Deletion of *comQXPA*
s8127	AAAAAGAATTCTTTCTCCTTGATCCGGACAG	Deletion of *comQXPA*
s8128	AAAAAGCATGCAGGCCACAACTTTAGCGTTG	Deletion of *comQXPA*
s8132	AAAAAGCATGCAATGGCGGAACAATTGTGAAC	Deletion of *comK*
s8172	AAAAAGGATCCGGTTGAATATTCTCTGACCCC	Deletion of *srfA* (*comS*)
s8175	AAAAAGGATCCTTCCGTTTGTGACAGCAGGA	Deletion of *srfA* (*comS*)
s8249	TCACTTCATTTAGAATTCCATATTGTCATACCTCCCCTA	Deletion of *srfA* (*comS*)
s8250	TATGACAATATGGAATTCTAAATGAAGTGATGAAAGGAGG	Deletion of *srfA* (*comS*)
s8251	TATTTTTCTAGAATTCCATATTATGGCCTCCATCCTT	Deletion of *comK*
s8252	GCCATAATATGGAATTCTAGAAAAATAGGAAGGAGCTGA	Deletion of *comK*
s8253	AAAAAGCATGCTCAGGTAAACTAACGGGTCG	Deletion of *comK*
s8475	AAAAAAGGATCCGGGCTATTTCTTGAGTTG	*trpC2* mutation
s8476	AAAAAAGGATCCGAAGCACGTTATGTTTG	*trpC2* mutation
s8633	ATTCCTCTACTTGAAGAGAATCAATAATAAAATCTTTTCT AAGTACAGGAA	*trpC2* mutation
s8634	TTCCTGTACTTAGAAAAGATTTTATTATTGATTCTCTT CAAGTAGAGGAAT	*trpC2* mutation
s8791	CCCCCGGATCCATGAGTGATCGTCAGGCAG	Deletion of *recA*
s8792	CCCCCGGATCCTTATTCTTCAAATTCGAGTTCTT	Deletion of *recA*

s8856	GGGGGGATATCGTGAAAAAAGTAATAGGTATTTAG	Integration of *comK*, *comS* (P_{licB})
s8857	GGGGGCTTAAGCAGAAAAAGGATGGAGGCCA	Integration of *comK*, *comS* (P_{licB})
s9092	CCCCCCGGCCGCGTACCTATTAATGTATCGTTTT	Cat to delete *recA*
s9093	CCCCCCGGCCGCCATAGTGACTGGCGATGC	Cat to delete *recA*
s9314	AAAAAGCATGCCGGACCCGCTCAAATGCT	Deletion of *degU*
s9315	AAAAAGAATTCGTCACAAGCCACGCCTCC	Deletion of *degU*
s9316	CCCCCGAATTCATGAGATAGTATAATAGGAGACT	Deletion of *degU*
s9317	CCCCCGCATGCCTCAAAAGGCACAATTACCTTA	Deletion of *degU*
s10446	ATGTTTGCAAAACGATTCAAAAC	Replacement of *amyE* by *spcR*
s10447	ATTAAAAAAATTATAAAATTGACTGCCGTAACTG	Replacement of *amyE* by *spcR*
s10448	GCAGTCAATTTTATAATTTTTTTAATCTGTTATTTAAATAGTT TATAGTTAAATTTAC	Replacement of *amyE* by *spcR*
s10449	CAAATTTTGTTGGCGAATGGCGATTTTCG	Replacement of *amyE* by *spcR*
s10450	GCCATTCGCCAACAAAATTGCCTGATGGC	Replacement of *amyE* by *spcR*
s10451	ATGGGGAAGAGAACCGCT	Replacement of *amyE* by *spcR*
s10452	TTGCAAACATATGGGGAAGAGAACCGCT	Replacement of amyE by *spcR*
s10453	TCTTCCCCATATGTTTGCAAAACGATTCAAAAC	Replacement of amyE by *spcR*
s10576	GGGGAACGGTATTAGGAAAAGGAGGTCAATCTATTGG	Integration of *dprA by* Gibson Assembly
s10577	TTTAGATTTATATTGGGTGAAAGGGTTCCGTATATTGAAC	Integration of *dprA by* Gibson Assembly
mrpS-A	TCTGGCTCTTGATAACGTTCCTTCTCTAGAGCCAGA	*mrpS* in pWAL275
mrpS-B	AGCTTCTGGCTCTAGAGAAGGAACGTTATCAAGAGCCAGAACG T	*mrpS* in pWAL275

Table S2. Further plasmids and strains used in this study.

Strain or plasmid	Genotype	Source, Reference or Construction
B. subtilis		
REG89	*trpC2, ΔmanPA::ermC,* P_{licB}-*comK-comS*	pREG37→REG1
REG159	*trpC2, ΔmanPA::ermC,* P_{licB}-*comK-comS, ΔcomK*	pREG13→REG89
**Plasmid**		
pJOE7330.2	*ori*$_{pUC18}$, *bla*, *ter-luc-ter*	This study
pJOE7393.1	*ori*$^{+}_{pUB110}$, *ori*$_{pUC18}$, *rep*$_{pUB110}$, *spcR*, *ter*-P_{mtlA}-*luc-ter*	This study
pKAM114	*ori*$^{+}_{pUB110}$, *ori*$_{pUC18}$, *rep*$_{pUB110}$, *spcR*, *ter*-P_{mtlA}-*eGFP-ter*	This study
pKAM160	*ori*$_{pBS71}$, *ori*$_{pUC18}$, *repA*$_{pBS72}$, *rop*, *spcR*, *ter*-P_{licB}-*lacZ-ter*	Heravi and Altenbuchner (2014)
pMW168.1	*ori*$^{+}_{pUB110}$, *ori*$_{pUC18}$, *rep*$_{pUB110}$, *spcR*, *ter-'manR*-P_{manP}-*eGFP-ter*	Wenzel et al. (2011)
pREG36	*ori*$_{pBS71}$, *ori*$_{pUC18}$, *repA*$_{pBS72}$, *rop*, *spcR*, *ter*-P_{licB} *-comKS-'lacZ*	This study
pREG37	*ori*$_{pUC18}$, *bla*, *yvcB*'-P_{licB}-*comK-comS-hisI-hisF*'	This study

Chemicals, enzymes and kits used for DNA manipulation

Unless otherwise specified, desired DNA fragments were amplified in PCR using genomic DNA of *B. subtilis* 168 and Phusion® HF DNA polymerase (Catalog no. #M530S; New England BioLabs®, Frankfurt am Main, Germany) on a PTC-200 Peltier Thermal Cycler (MJ Research). Chromosomal DNA was isolated from *B. subtilis* with DNeasy® Blood & Tissue Kit (Cat no. 69506; Qiagen, Hilden, Germany). Plasmid DNA was isolated from *E. coli* using "innuPREP Plasmid mini Kit" (Analytic Jena AG, Jena, Germany). Concentrations of plasmid and chromosomal DNA were measured at 260 nm using a NanoPhotometer (IMPLEN GmbH, Munich, Germany). Restriction enzymes were purchased from New England BioLabs® (Frankfurt am Main, Germany). T4 DNA Ligase was provided by Thermo Fisher Scientific Inc. (St. Leon-Rot, Germany). Digested DNA fragments from agarose gel and amplified DNAs in PCRs were isolated with "NucleoSpin® Gel and PCR Clean-up" kit (Machery-Nagel, Düren, Germany). DNA samples were sequenced by GATC Biotech (Konstanz, Germany).

Construction of plasmid pKAM180

Each of the P_{mtlA} and *luc* (luciferase originated from *Photinus pyralis*) DNA fragments was derived from different plasmids. Amplification of the *luc* gene was carried out in a PCR using oligonucleotides s7705 and s7706 using pGL2-Basic (Promega, USA) as the template. The 1.6 kb PCR product was then inserted into pJOE4786.1 via *Sma*I site to construct pJOE7330.2. Next, the *luc* gene was cut from pJOE7330.2 by *Sna*BI and *Nde*I and inserted into pKAM114 (containing P_{mtlA}-*eGFP*) cut with *Sma*I and *Nde*I to construct pJOE7393.1. Plasmid pKAM114 was a derivative of the pUB110-based plasmid pMW168.1. To create pKAM114, the P_{mtlA} DNA fragment was amplified in a PCR using s7355 and s7356. The PCR product (P_{mtlA}) was then digested with *Age*I and *Bam*HI and inserted into pMW168.1 cut with the same enzymes.

Afterwards, the P_{mtlA}-*luc* cassette on pJOE7393.1 was cut by *Age*I and *Pme*I and inserted into pHM31 digested with *Xma*I and *Nae*I to create pKAM180.

Construction of plasmid pREG37

To integrate the P_{licB}-*comKS* cassette into the *B. subtilis* chromosome, *comKS* DNA was amplified in a PCR using oligonucleotides s8856-s8857 and pJOE7361.1 as the template. Next, *lacZ* was cut from pKAM160 (P_{licB}-*lacZ*) with *Afl*II and *Eco*RV and *comKS* DNA was inserted instead (pREG36). The P_{licB}-*comKS* cassette was then cut out from pREG36 by *Nhe*I and inserted into pHM31 through *Nhe*I sites creating pREG37.

Construction of REG89 and REG159 strains

Strain REG89 was constructed after transformation of REG1 with pREG37. The selection of the transformants was carried out on minimal medium without histidine as described before (Motejadded and Altenbuchner, 2007). REG159 was created by transformation of REG89 with pREG13. The transformation procedure and mutant selection was carried out according to Wenzel and Altenbuchner (2015).

Results

Induction of competence by expression of *comK* and *comS* under the control of P_{licB}

The high level of REG19 competence was dependent on the presence of natural *comK*. To see if another, and maybe, stronger promoter would overcome this dependency, the *B. subtilis* mannitol inducible promoter (P_{mtlA}) in REG19 was replaced by the cellobiose inducible promoter P_{licB} (REG89). The competence of the strains REG19 and REG89 was induced by mannitol and cellobiose, respectively, and the cells transformed by addition of pWAL275 dimer DNA. The results are shown in **Figure S1**. The exchange of the promoter resulted in a 2.5-fold increase of transformation efficiency. Obviously, more cells became competent, when *comK* and *comS* were

expressed by P_{licB}. Even the transformation rate of uninduced REG89 was quite high compared with uninduced REG19. In the next step, the original *comK* gene was deleted from REG89 and the transformation efficiency of the new strain REG159 (REG89 Δ*comK*) tested by the addition of pWaL275 dimer DNA. Now, deletion of the original *comK* resulted in only a slight (0.8-fold) loss of transformation efficiency (**Figure S1**). Interestingly, when REG159 was not induced by cellobiose, the transformation efficiency was still very high with 1.4×10^4 colonies per ml.

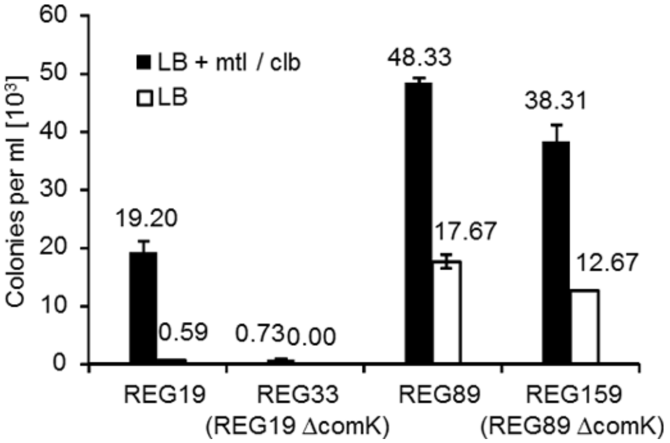

Figure S1 Comparison of the transformation efficiency of strains REG19 (P_{mtlA}-*comKS*), REG89 (P_{licB}-*comKS*) and influence of the deletion of original *comK* in REG89 (REG159). All strains were transformed with 100 ng of the pWaL275 dimers in LB with(out) mannitol (mtl) or with(out) cellobiose (clb). Error bars represent standard deviation from the mean value between triplicate experiments.

References

Heravi, K.M., and Altenbuchner, J. (2014). Regulation of the *Bacillus subtilis* mannitol utilization genes: promoter structure and transcriptional activation by the wild-type regulator (MtlR) and its mutants. *Microbiology* 160**,** 91-101. doi: 10.1099/mic.0.071233-0.

Motejadded, H., and Altenbuchner, J. (2007). Integration of a lipase gene into the Bacillus subtilis chromosome: Recombinant strains without antibiotic resistance marker. *Iran J Biotechnol*.

Wenzel, M., and Altenbuchner, J. (2015). Development of a markerless gene deletion system for *Bacillus subtilis* based on the mannose phosphoenolpyruvate-dependent phosphotransferase system. *Microbiology*. doi: 10.1099/mic.0.000150.

Wenzel, M., Müller, A., Siemann-Herzberg, M., and Altenbuchner, J. (2011). Self-inducible *Bacillus subtilis* expression system for reliable and inexpensive protein production by high-cell-density fermentation. *Appl Environ Microbiol* 77**,** 6419-6425. doi: 10.1128/AEM.05219-11.
